# Supplementary material for: Expression, Localization, and Activity of the Aryl Hydrocarbon Receptor in the Human Placenta
Source: Int J Mol Sci. 2018 Nov 27;19(12):3762. doi: 10.3390/ijms19123762 (PMC6321474; doi:10.3390/ijms19123762)
Supplement: Supplementary file 1 [file ijms-19-03762-s001.pdf]

Supplementary Materials

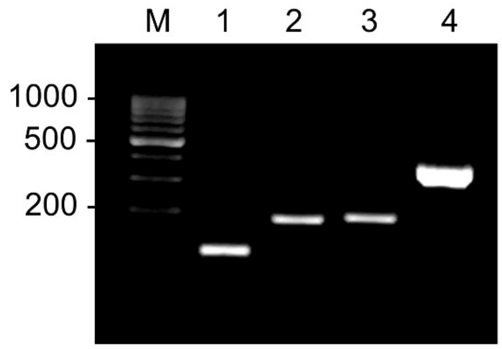

Figure S1. Test of the designed gene-specific primers. Total RNA extracted from human primary villous cytotrophoblasts was analyzed by RT-PCR using primers for *AhR* (lane 1), *AhRR* (lane 2), *ARNT* (lane 3) and *β-actin* (lane 4). DNA molecular size marker (100bp ladder) was deposed in lane M.

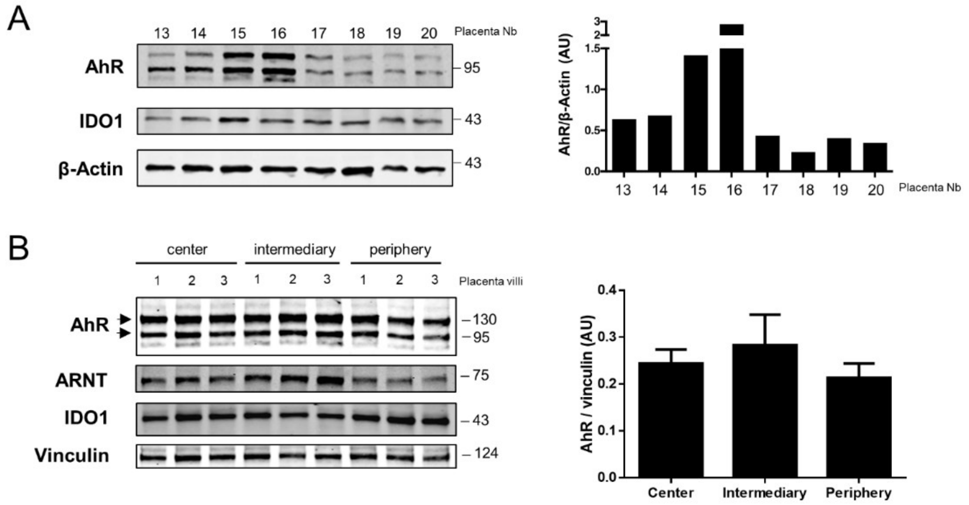

Figure S2. Expression of the AhR and IDO1 proteins in different sections of the placenta at term. (A) Immunoblotting of AhR and IDO1 in total protein extracts of villi from 8 individual placentae after migration on SDS-PAGE. β-Actin is used as loading control. The quantification of total AhR-bands (2 bands) normalized to β-Actin is shown (right plot). Placentae number 13, 15, 17 and 18 respectively correspond to placentae number 9, 10 11 and 12 on the figure 1C. **(B)** Three distinct chorionic villi have been harvested from different areas of placentae from the center (close to umbilical cord), the inner side (intermediary) and at the exterior (periphery). Total proteins were extracted and subjected to SDS-PAGE under reducing conditions, and immunobloted with anti-AhR, anti-ARNT, anti-IDO1 and anti-vinculin antibodies. The bar graph represents the total amount of AhR protein (2 bands) relative to vinculin levels determined by quantification of immunoblots using the Odyssey System Imager. The means and standard deviations of 3 villi are shown.

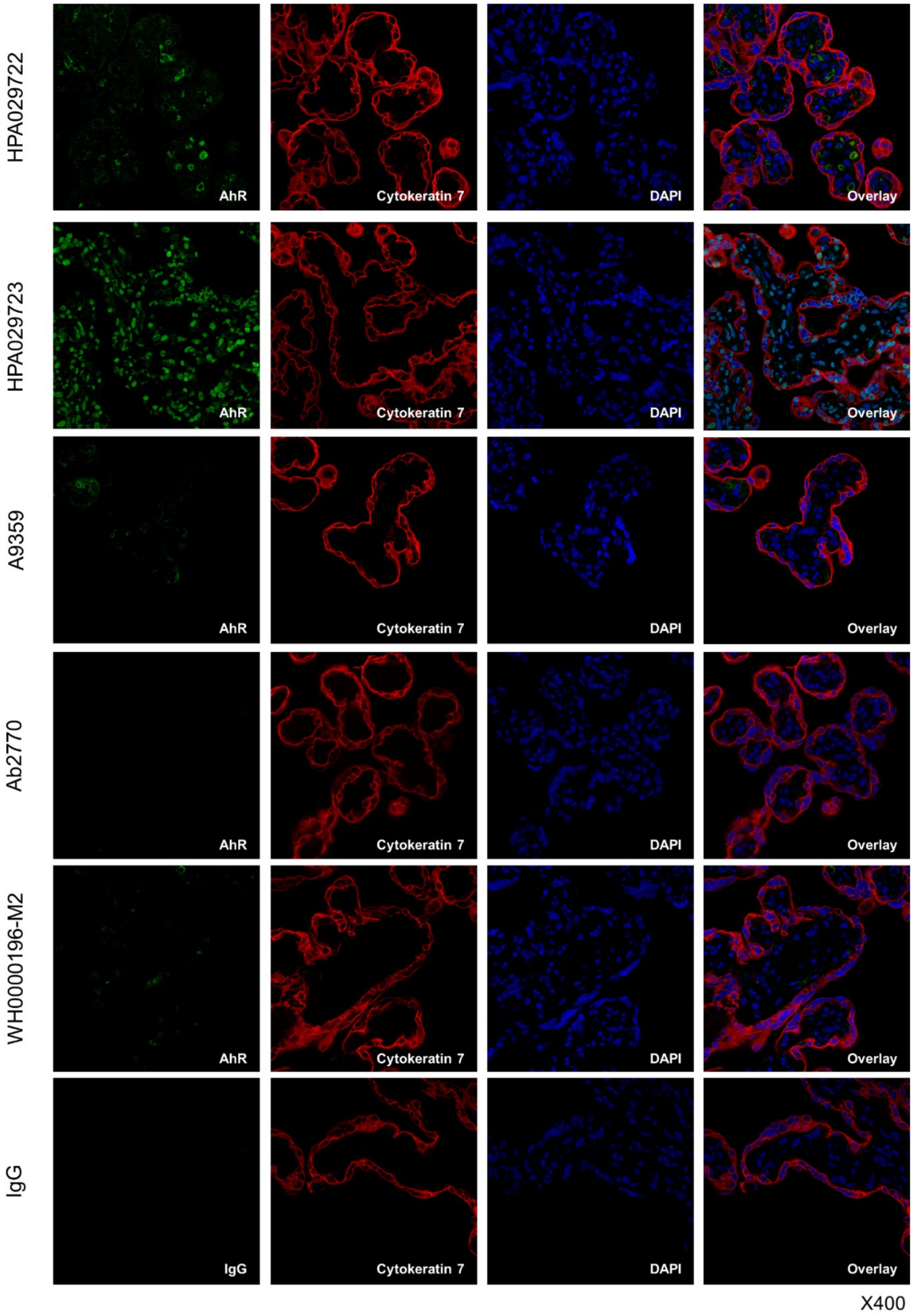

Figure S3. Test of several anti-AhR antibodies for AhR-staining on placental villi. Placental villi were fixed in 4% PFA, included in agarose, sliced and permeabilized with 0.5% Triton. Images are of confocal microscopy, AhR (green) was stained with the indicated antibody and non-specific IgG was used as a background control. Trophoblasts were stained by cytokeratin-7 (red) and nuclei were visualized in blue (DAPI staining). Overlay images are in the right panel.

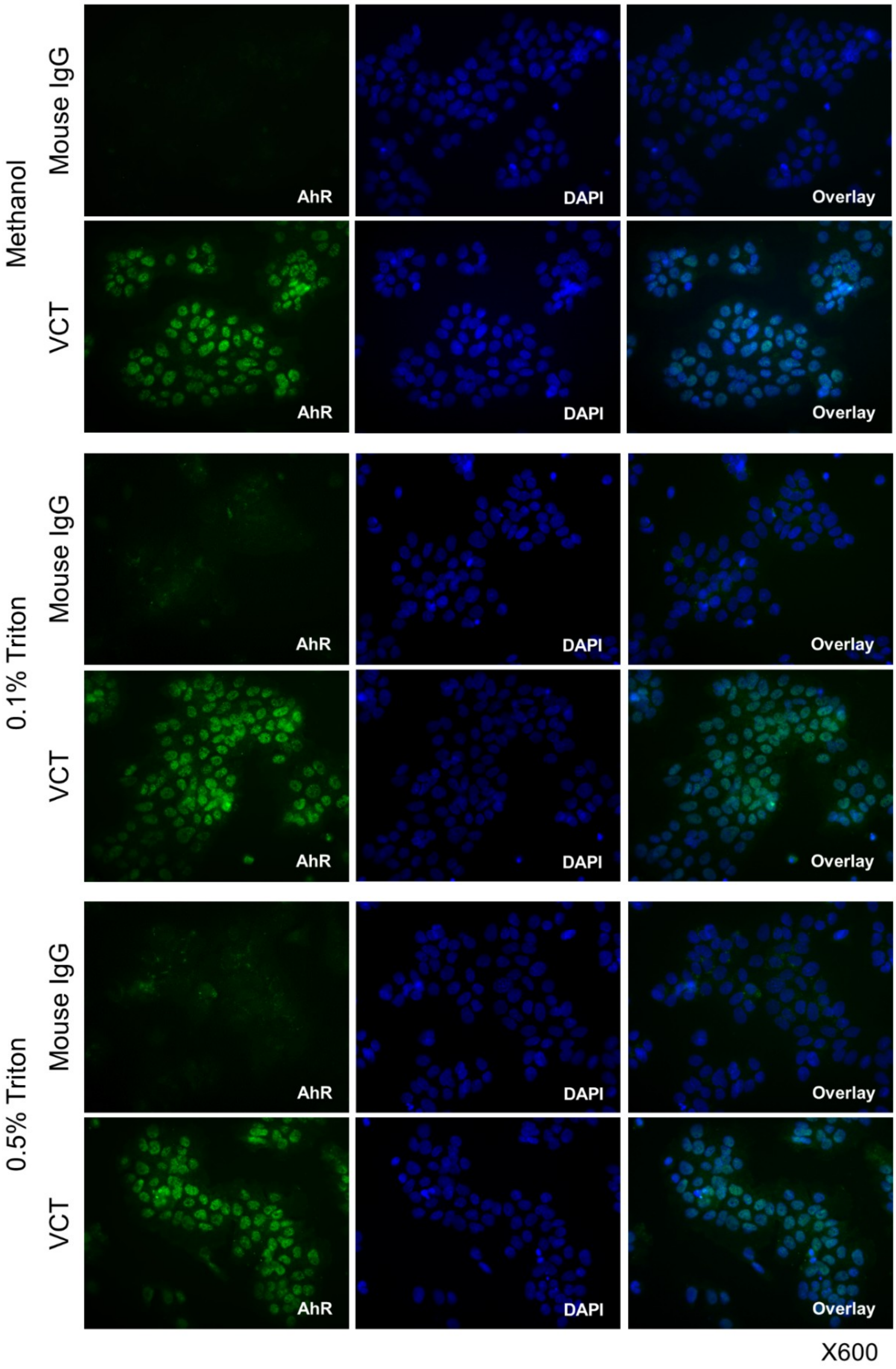

Figure S4. The optimization of the fixation/permeabilization protocol for AhR-staining in villous cytotrophoblasts (VCT) cells. Human primary cytotrophoblasts after 24h of culture were fixed either with PFA or methanol and then cell membrane were permeabilized using 0.1% Triton, 0.5% Triton or methanol (as indicated). Images are of epifluorescence microscopy, AhR was stained with the WH0000196-M2 antibody and IgG was used as background control. Nuclei are visualized in blue (DAPI staining). Overlay images are in right panel.

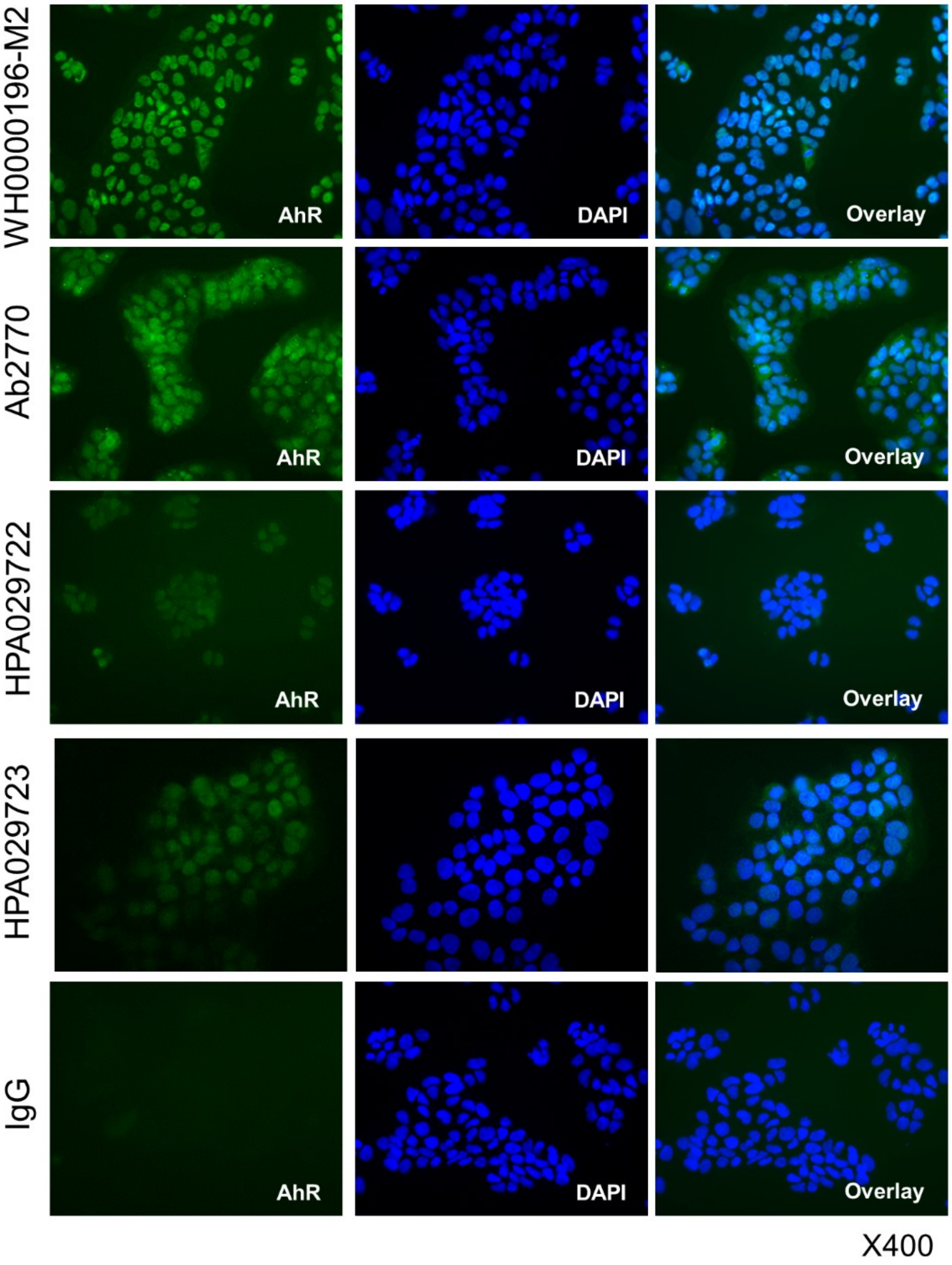

Figure S5. Test of several anti-AhR antibodies on BeWo cells. Epifluorescence microscopy of AhR (green) using the WH0000196-M2 (Sigma), Ab2770 (Abcam), HPA029722 (Sigma) and HPA029723 (Sigma) antibodies. Nuclei are shown in blue after DAPI staining and the overlay images are shown in the right panel.

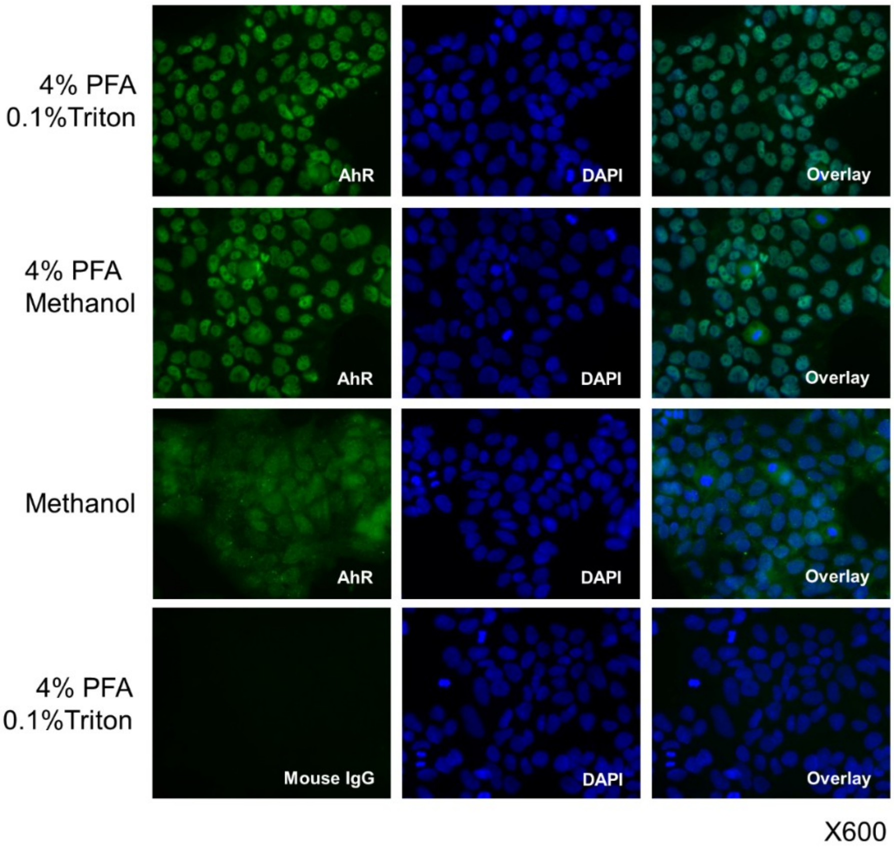

Figure S6. Optimization of the fixation/permeabilization protocol for AhR-staining in BeWo cells. BeWo cells were fixed either with PFA or methanol and then cell membrane were permeabilized using 0.1% Triton or methanol (as indicated). Images are of epifluorescence microscopy where AhR was stained with the WH0000196-M2 antibody or mouse non-specific IgG was used as a background control. Nuclei are visualized in blue (DAPI staining). Overlay images are in right panel.

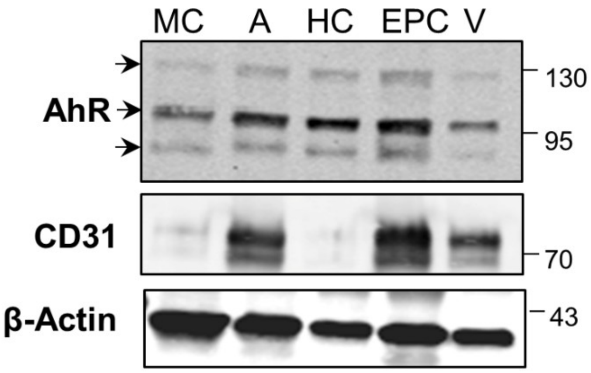

Figure S7. Detection of the AhR in placental cells. Western blotting of total protein from villous mesenchymal cells (MC), umbilical artery endothelial cells (A), collagenous extract from umbilical cord (HC) composed of choriotrophoblasts and mesenchymal cells, endothelial progenitor cells (EPC) and umbilical vein endothelial cells (V). Immunoblotting was carried out with anti-AhR antibody. Extracts were tested with anti-CD31 as an endothelial marker and β-Actin was used as loading control.
